# 4,5-Diamino­benzene-1,2-dicarbonitrile

**DOI:** 10.1107/S1600536809008733

**Published:** 2009-03-25

**Authors:** Xiuwen Zhang, Wei Wang, Jianzhuang Jiang, Zhonghai Ni

**Affiliations:** a School of Chemistry & Chemical Engineering, Shandong University, Jinan 250100, People’s Republic of China

## Abstract

The mol­ecular skeleton of the title mol­ecule, C_8_H_6_N_4_, is essentially planar [maximum deviation from the mean plane of 0.037 (2) Å]. All N atoms are involved in the formation of inter­molecular N—H⋯N hydrogen bonds. The crystal packing exhibits also dipole–dipole inter­actions between the cyano groups of neighbouring mol­ecules [C⋯C 3.473 (2) Å].

## Related literature

For details of the synthesis, see: Cheeseman (1962 ▶); Mitzel *et al.* (2003 ▶). For applications of diamido compounds, see: Rusanova *et al.* (2002 ▶); Youngblood (2006 ▶). For a related crystal structure, see: Zhang & Lu (2007 ▶).
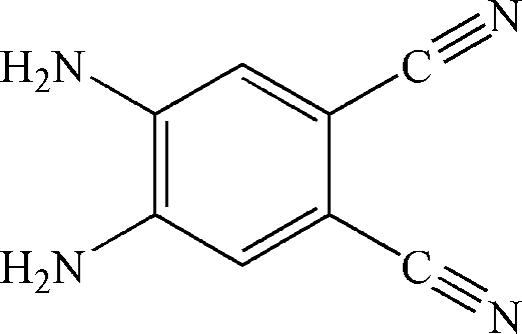


## Experimental

### 

#### Crystal data

C_8_H_6_N_4_
*M* *_r_* = 158.17
Monoclinic, 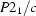

*a* = 8.2966 (11) Å
*b* = 17.100 (2) Å
*c* = 5.5295 (7) Å
β = 102.256 (2)°
*V* = 766.60 (17) Å^3^
*Z* = 4
Mo *K*α radiation
μ = 0.09 mm^−1^
*T* = 273 K
0.20 × 0.18 × 0.14 mm

#### Data collection

Bruker APEXII CCD area-detector diffractometer
Absorption correction: multi-scan (*SADABS*; Sheldrick, 2003 ▶) *T*_min_ = 0.980, *T*_max_ = 0.988
4031 measured reflections
1502 independent reflections
1201 reflections with *I* > 2σ(*I*)
*R*_int_ = 0.018

#### Refinement

*R*[*F* ^2^ > 2σ(*F* ^2^)] = 0.041
*wR*(*F* ^2^) = 0.146
*S* = 0.95
1502 reflections
109 parameters
H-atom parameters constrained
Δρ_max_ = 0.25 e Å^−3^
Δρ_min_ = −0.21 e Å^−3^

### 

Data collection: *APEX2* (Bruker, 2004 ▶); cell refinement: *SAINT-Plus* (Bruker, 2001 ▶); data reduction: *SAINT-Plus*; program(s) used to solve structure: *SHELXS97* (Sheldrick, 2008 ▶); program(s) used to refine structure: *SHELXL97* (Sheldrick, 2008 ▶); molecular graphics: *XP* in *SHELXTL* (Sheldrick, 2008 ▶); software used to prepare material for publication: *XP* in *SHELXTL*.

## Supplementary Material

Crystal structure: contains datablocks global, I. DOI: 10.1107/S1600536809008733/cv2527sup1.cif

Structure factors: contains datablocks I. DOI: 10.1107/S1600536809008733/cv2527Isup2.hkl

Additional supplementary materials:  crystallographic information; 3D view; checkCIF report

## Figures and Tables

**Table 1:** Hydrogen-bond geometry (Å, °)

*D*—H⋯*A*	*D*—H	H⋯*A*	*D*⋯*A*	*D*—H⋯*A*
N2—H2*A*⋯N3^i^	0.86	2.47	3.283 (2)	158
N2—H2*B*⋯N4^ii^	0.86	2.37	3.225 (2)	171
N1—H1*A*⋯N1^iii^	0.86	2.52	3.3729 (16)	169
N1—H1*B*⋯N4^ii^	0.86	2.34	3.188 (2)	171

Symmetry codes: (i) 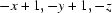; (ii) 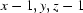; (iii) 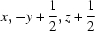.

**Fig. 1.**
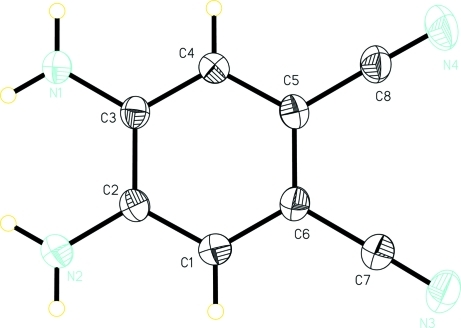
A view of (I) with the unique atom-labelling scheme. Displacement ellipsoids are drawn at the 30% probability level.

## supplementary crystallographic information

### Comment

Diamido compounds have been paid much attention becuase of their wide application in the preparation of Schiff bases and other organic ligands. On the other hand, dicyano compounds have been widely used to synthesize many useful materials such as phthalocyanine dyes. Very recently, organic ligands with differernt functional groups have attracted intense interest in the design and synthesis of functional materials, among which the title compound (I) as an very interesting small organic bifunctional precursor have been synthesied and employed to design and synthesize phthalocyanine compounds (Rusanova *et al.*, 2002; Mitzel *et al.*, 2003; Youngblood *et al.*, 2006). Herein, we report its crystal structure (Fig. 1).

The whole molecular structure of (I) is essentially planar with the largest deviation value of 0.037 (2) Å from the mean plane. The cyano groups bond lengths are 1.140 (2) and 1.142 (2) Å, respectively, which are similar to those in cyano-substituted organic ligands (Zhang *et al.*, 2007).

In the crystal, the molecules are linked by four different N···H—N intermolecular hydrogen bonds (Table 2) between primary amido hydrogen atoms and amido and cyano nitrogen atoms. In additon, the crystal packing exhibits dipole-dipole interactions between the cyano groups of neighbouring molecules proved by short distance C6···C7(-*x* + 1, -*y* + 1, -*z* + 1) of 3.473 (2) Å (Table 1).

### Experimental

The title compound 4,5-diamido-1,2-dicyanobenzene was prepared by four steps reaction from the starting material 1,2-diamidobenzene according to the method reported in the literature (Cheeseman, 1962; Mitzel *et al.*, 2003). A solid of 4,5-diamido-1,2-dicyanobenzene (0.5 mmol) was added to the acetone solution (8 ml). The solution was slowly evaporated to generate white block single crystals suitable for X-ray diffraction analysis. Elemental analysis [found (calculated)] for C_8_H_6_N_4_: C 60.63 (60.75), H 3.77 (3.82), N 35.36% (35.42%).

### Refinement

All H-atoms were geometrically positioned (C—H 0.93 Å, N—H = 0.86 Å), and refined as riding, with *U*_iso_ = 1.2*U*_eq_ (C, N).

### Crystal data

**Table:**

C_8_H_6_N_4_	*F*(000) = 328
*M**_r_* = 158.17	*D*_x_ = 1.370 Mg m^−^^3^
Monoclinic, *P*2_1_/*c*	Mo *K*α radiation, λ = 0.71073 Å
Hall symbol: -P 2ybc	Cell parameters from 1651 reflections
*a* = 8.2966 (11) Å	θ = 2.5–26.5°
*b* = 17.100 (2) Å	µ = 0.09 mm^−^^1^
*c* = 5.5295 (7) Å	*T* = 273 K
β = 102.256 (2)°	Block, white
*V* = 766.60 (17) Å^3^	0.20 × 0.18 × 0.14 mm
*Z* = 4	

### Data collection

**Table:**

Bruker APEXII CCD area-detector diffractometer	1502 independent reflections
Radiation source: fine-focus sealed tube	1201 reflections with *I* > 2σ(*I*)
graphite	*R*_int_ = 0.018
Detector resolution: 0 pixels mm^-1^	θ_max_ = 26.0°, θ_min_ = 2.5°
φ and ω scans	*h* = −10→9
Absorption correction: multi-scan (*SADABS*; Sheldrick, 2003)	*k* = −18→21
*T*_min_ = 0.980, *T*_max_ = 0.988	*l* = −5→6
4031 measured reflections	

### Refinement

**Table:**

Refinement on *F*^2^	Primary atom site location: structure-invariant direct methods
Least-squares matrix: full	Secondary atom site location: difference Fourier map
*R*[*F*^2^ > 2σ(*F*^2^)] = 0.041	Hydrogen site location: inferred from neighbouring sites
*wR*(*F*^2^) = 0.146	H-atom parameters constrained
*S* = 0.95	*w* = 1/[σ^2^(*F*_o_^2^) + (0.1*P*)^2^ + 0.1224*P*] where *P* = (*F*_o_^2^ + 2*F*_c_^2^)/3
1502 reflections	(Δ/σ)_max_ < 0.001
109 parameters	Δρ_max_ = 0.25 e Å^−^^3^
0 restraints	Δρ_min_ = −0.21 e Å^−^^3^

### Special details

**Table:**

Geometry. All e.s.d.'s (except the e.s.d. in the dihedral angle between two l.s. planes) are estimated using the full covariance matrix. The cell e.s.d.'s are taken into account individually in the estimation of e.s.d.'s in distances, angles and torsion angles; correlations between e.s.d.'s in cell parameters are only used when they are defined by crystal symmetry. An approximate (isotropic) treatment of cell e.s.d.'s is used for estimating e.s.d.'s involving l.s. planes.
Refinement. Refinement of *F*^2^ against ALL reflections. The weighted *R*-factor *wR* and goodness of fit *S* are based on *F*^2^, conventional *R*-factors *R* are based on *F*, with *F* set to zero for negative *F*^2^. The threshold expression of *F*^2^ > σ(*F*^2^) is used only for calculating *R*-factors(gt) *etc*. and is not relevant to the choice of reflections for refinement. *R*-factors based on *F*^2^ are statistically about twice as large as those based on *F*, and *R*- factors based on ALL data will be even larger.

### Fractional atomic coordinates and isotropic or equivalent isotropic displacement parameters (Å^2^)

**Table:**

	*x*	*y*	*z*	*U*_iso_*/*U*_eq_	
N1	0.03342 (15)	0.30649 (8)	0.3604 (3)	0.0488 (4)	
H1A	0.0217	0.2745	0.4754	0.059*	
H1B	−0.0488	0.3167	0.2412	0.059*	
N2	0.07879 (17)	0.40517 (9)	−0.0247 (3)	0.0548 (4)	
H2A	0.0940	0.4338	−0.1457	0.066*	
H2B	−0.0173	0.3862	−0.0256	0.066*	
N3	0.78147 (18)	0.46391 (10)	0.3631 (3)	0.0667 (5)	
N4	0.7218 (2)	0.32364 (11)	0.9135 (3)	0.0736 (5)	
C1	0.36358 (19)	0.42015 (9)	0.1736 (3)	0.0449 (4)	
H1C	0.3794	0.4522	0.0448	0.054*	
C2	0.20725 (18)	0.38986 (9)	0.1690 (3)	0.0396 (4)	
C3	0.18407 (17)	0.34143 (8)	0.3672 (3)	0.0378 (4)	
C4	0.31750 (18)	0.32537 (8)	0.5584 (3)	0.0409 (4)	
H4A	0.3022	0.2937	0.6886	0.049*	
C5	0.47413 (18)	0.35546 (9)	0.5606 (3)	0.0411 (4)	
C6	0.49666 (18)	0.40366 (9)	0.3661 (3)	0.0417 (4)	
C7	0.6563 (2)	0.43676 (10)	0.3652 (3)	0.0492 (4)	
C8	0.6111 (2)	0.33725 (10)	0.7576 (3)	0.0506 (4)	

### Atomic displacement parameters (Å^2^)

**Table:**

	*U*^11^	*U*^22^	*U*^33^	*U*^12^	*U*^13^	*U*^23^
N1	0.0348 (7)	0.0570 (8)	0.0518 (8)	−0.0050 (6)	0.0027 (6)	0.0110 (6)
N2	0.0419 (8)	0.0688 (9)	0.0497 (8)	−0.0017 (6)	0.0008 (6)	0.0155 (7)
N3	0.0443 (9)	0.0755 (11)	0.0815 (12)	−0.0086 (7)	0.0157 (8)	0.0026 (8)
N4	0.0496 (9)	0.1007 (14)	0.0603 (10)	0.0000 (9)	−0.0112 (8)	0.0071 (9)
C1	0.0428 (9)	0.0485 (9)	0.0440 (9)	−0.0010 (7)	0.0103 (7)	0.0055 (7)
C2	0.0370 (8)	0.0410 (8)	0.0394 (8)	0.0035 (6)	0.0048 (6)	−0.0004 (6)
C3	0.0342 (7)	0.0375 (7)	0.0415 (8)	0.0009 (5)	0.0072 (6)	−0.0031 (6)
C4	0.0394 (9)	0.0446 (8)	0.0376 (8)	−0.0009 (6)	0.0054 (6)	0.0035 (6)
C5	0.0366 (8)	0.0448 (8)	0.0394 (8)	0.0008 (6)	0.0028 (6)	−0.0033 (6)
C6	0.0349 (8)	0.0463 (8)	0.0437 (8)	−0.0010 (6)	0.0080 (6)	−0.0030 (6)
C7	0.0425 (9)	0.0529 (9)	0.0527 (10)	−0.0008 (7)	0.0113 (7)	0.0011 (7)
C8	0.0399 (9)	0.0610 (10)	0.0480 (9)	−0.0040 (7)	0.0028 (7)	−0.0008 (7)

### Geometric parameters (Å, °)

**Table:**

N1—C3	1.3787 (19)	C1—C2	1.392 (2)
N1—H1A	0.8600	C1—H1C	0.9300
N1—H1B	0.8600	C2—C3	1.419 (2)
N2—C2	1.366 (2)	C3—C4	1.387 (2)
N2—H2A	0.8600	C4—C5	1.395 (2)
N2—H2B	0.8600	C4—H4A	0.9300
N3—C7	1.140 (2)	C5—C6	1.399 (2)
N4—C8	1.142 (2)	C5—C8	1.432 (2)
C1—C6	1.390 (2)	C6—C7	1.441 (2)
			
C6···C7^i^	3.473 (2)		
			
C3—N1—H1A	120.0	N1—C3—C2	120.18 (13)
C3—N1—H1B	120.0	C4—C3—C2	119.18 (13)
H1A—N1—H1B	120.0	C3—C4—C5	121.64 (14)
C2—N2—H2A	120.0	C3—C4—H4A	119.2
C2—N2—H2B	120.0	C5—C4—H4A	119.2
H2A—N2—H2B	120.0	C4—C5—C6	119.15 (13)
C6—C1—C2	121.61 (14)	C4—C5—C8	120.90 (14)
C6—C1—H1C	119.2	C6—C5—C8	119.95 (13)
C2—C1—H1C	119.2	C1—C6—C5	119.61 (13)
N2—C2—C1	120.78 (14)	C1—C6—C7	119.94 (14)
N2—C2—C3	120.42 (14)	C5—C6—C7	120.45 (14)
C1—C2—C3	118.80 (14)	N3—C7—C6	179.01 (18)
N1—C3—C4	120.48 (14)	N4—C8—C5	178.92 (19)

Symmetry codes: (i) −*x*+1, −*y*+1, −*z*+1.

### Hydrogen-bond geometry (Å, °)

**Table:**

*D*—H···*A*	*D*—H	H···*A*	*D*···*A*	*D*—H···*A*
N2—H2A···N3^ii^	0.86	2.47	3.283 (2)	158
N2—H2B···N4^iii^	0.86	2.37	3.225 (2)	171
N1—H1A···N1^iv^	0.86	2.52	3.3729 (16)	169
N1—H1B···N4^iii^	0.86	2.34	3.188 (2)	171

Symmetry codes: (ii) −*x*+1, −*y*+1, −*z*; (iii) *x*−1, *y*, *z*−1; (iv) *x*, −*y*+1/2, *z*+1/2.
